# Trends in Enrollment in Employer-Sponsored Health Insurance in the US Before and During the COVID-19 Pandemic, January 2019 to June 2021

**DOI:** 10.1001/jamanetworkopen.2022.34174

**Published:** 2022-09-30

**Authors:** Ravi B. Parikh, Amol S. Navathe, Yueming Zhao, David Pagnotti, Ezekiel J. Emanuel

**Affiliations:** 1 Department of Medical Ethics and Health Policy, University of Pennsylvania, Philadelphia; 2 Department of Medicine, Perelman School of Medicine, University of Pennsylvania, Philadelphia; 3 Leonard Davis Institute of Health Economics, University of Pennsylvania, Philadelphia; 4 Corporal Michael J. Crescenz VA Medical Center, Philadelphia, Pennsylvania

## Abstract

This cross-sectional study compares trends in employer-sponsored health insurance coverage in the US before and during the COVID-19 pandemic.

## Introduction

Employer-sponsored health insurance (ESI) covers approximately 160 million US individuals.^1^ Decreased employment during the COVID-19 pandemic may have led to loss of ESI. We assessed trends in ESI coverage among members of a large group of US health plans.

## Methods

This cross-sectional study analyzed data from a group of US commercial health plans operating in 50 states for members from large and small employers who were enrolled between January 1, 2019, and June 30, 2021. We defined enrollment as the number of eligible members enrolled in a plan at any point in each month. The unit of analysis was at the hospital referral region (HRR)–month level.^2^ The University of Pennsylvania institutional review board declared the study exempt and waived informed consent owing to use of deidentified historical data. We followed the STROBE guideline.

We analyzed COVID-19–associated changes in overall enrollment during 2020 and 2021 vs 2019 using a linear model with month and HRR fixed effects. Covariates included age, self-reported sex, Elixhauser comorbidity index,^3^ income, and educational level (latter 2 identified using American Community Survey data [matched by zip code]). We defined January 1, 2019, to March 31, 2020, as the pre-COVID-19 period and April 1 to December 31, 2020, and January 1 to June 30, 2021, as exposure periods. We analyzed enrollment trends in prespecified demographic (sex), location (urban vs rural), employer size (small vs large [<500 vs ≥500 employees]), and geographic (Medicaid expansion vs nonexpansion state) groups. To compare enrollment trends across subgroups, we used separate models indexing absolute enrollment numbers in preexposure and exposure periods as the ratio to enrollment in December 2019 and using subgroup × exposure period interaction terms. We conducted a sensitivity analysis focusing on main subscribers of the plan. Two-sided *P* < .05 was significant. Analyses were conducted using Stata MP, version 14.1.

## Results

The sample included more than 18 million unique individuals (mean [SD] age, 33.6 [19.2] years; 49.8% female; 50.2% male; 3.1% lived in a rural domicile). There were no significant differences in baseline characteristics across periods. Overall enrollment was 13 346 213 in January 2019, 14 083 411 in January 2020, 13 586 944 in December 2020, and 13 359 615 in June 2021, a decrease of 723 796 members (5.1%) from January 2020 to June 2021 (Figure). Mean adjusted monthly enrollment per HRR decreased from 45 852 before COVID-19 to 44 851 during the 2020 COVID-19 period (adjusted change, −1258; 95% CI, –1504 to −1011) (Table) and to 44 516 during the 2021 COVID-19 period (adjusted change, −1968; 95% CI, −2204 to −1733). Decreases were disproportionately greater among females and members with small employers. We did not observe meaningful differences in urban vs rural subgroups. Results of the sensitivity analysis were consistent.

**Figure.**
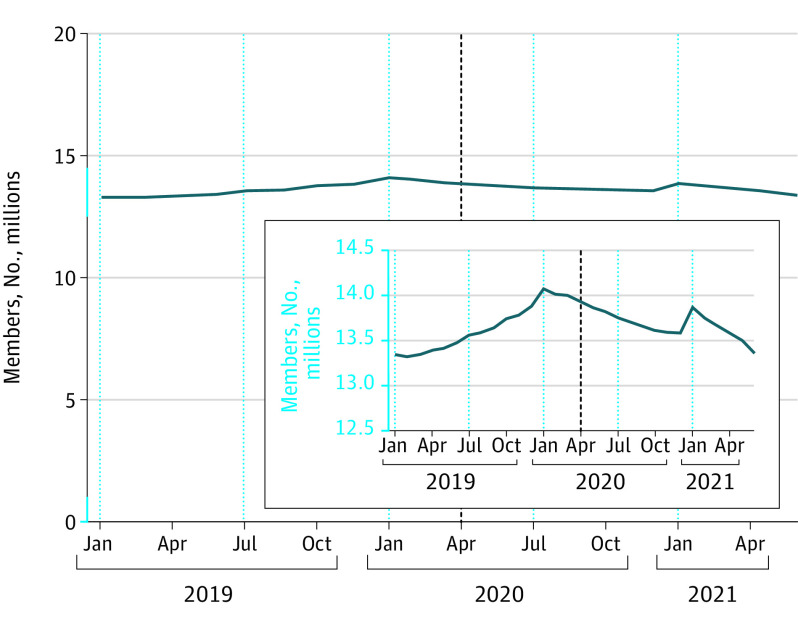
Trends in Commercial Insurance Coverage From January 2019 to June 2021 The inset has a curtailed y-axis to better show decreases in enrollment during the COVID-19 pandemic period. Increases in January represent new enrollees at the beginning of a calendar year.

**Table.** Adjusted Change in Enrollment Over Time

	2019		2020		January to June 2021	Adjusted change^a^	*P* value	Adjusted change^b^	*P* value
	January to March	April to December	January to March	April to December					
**Enrollment** ^c^	44 081 (43 939 to 44 223)	44 324 (44 232 to 44 415)	45 852 (45 686 to 46 018)	44 851 (44 755 to 44 947)	44 516 (44 398 to 44 633)	–1258 (–1504 to – 1011)	<.001	–1968 (–2204 to –1733)	.001

^a^ Compares the April to December 2020 period with the prepandemic baseline (January 2019 to March 2020).

^b^ Compares the January to June 2021 period with the prepandemic baseline (January 2019 to March 2020).

^c^ Cell values represent enrollment at the hospital referral region–month level.

## Discussion

Enrollment in ESI decreased by 723 796 members throughout the COVID-19 pandemic period and did not recover to pre-COVID-19 levels by mid-2021, consistent with previous evidence of decreased insurance coverage early in the pandemic estimated by studying 2021 trends and heterogeneity in enrollment decreases in the commercially insured population.^4^ Our findings have 3 implications. First, ESI coverage decreased even as US unemployment decreased from 14.7% in April 2020 to 6.7% in December 2020.^4^ Second, coverage losses were higher among individuals working for small employers, reflecting the pandemic’s disproportionate effects on small business employment.^5^ Third, female members had greater enrollment decreases, consistent with evidence of decreases in the female labor force during the pandemic.^6^ Study limitations include the focus on a continuously enrolled, commercially insured population, which prevented measurement of changes in enrollment trends across other commercial plans, and the inability to attribute disenrollment to job loss vs voluntary withdrawal. By limiting to continuously participating employers, we were unable to measure employers that went out of business during the pandemic. Given that enrollment decreased through 2021, examination of the pace of commercial insurance enrollment in 2022 is critical.
